# A structural deep network embedding model for predicting associations between miRNA and disease based on molecular association network

**DOI:** 10.1038/s41598-021-91991-w

**Published:** 2021-06-16

**Authors:** Hao-Yuan Li, Hai-Yan Chen, Lei Wang, Shen-Jian Song, Zhu-Hong You, Xin Yan, Jin-Qian Yu

**Affiliations:** 1 School of Computer Science and Technology, China University of Mining and Technology, Xuzhou, 221116 China; 2 Xinjiang Autonomous Region tax Service, State Taxation Administration, Urumqi, 830011 China; 3 Xinjiang Technical Institutes of Physics and Chemistry, Chinese Academy of Sciences, Urumqi, 830011 China; 4 Science & Technology Department of Xinjiang Uygur Autonomous Region, Urumqi, 830011 China

**Keywords:** Computational biology and bioinformatics, Computational models, Machine learning

## Abstract

Previous studies indicated that miRNA plays an important role in human biological processes especially in the field of diseases. However, constrained by biotechnology, only a small part of the miRNA-disease associations has been verified by biological experiment. This impel that more and more researchers pay attention to develop efficient and high-precision computational methods for predicting the potential miRNA-disease associations. Based on the assumption that molecules are related to each other in human physiological processes, we developed a novel structural deep network embedding model (SDNE-MDA) for predicting miRNA-disease association using molecular associations network. Specifically, the SDNE-MDA model first integrating miRNA attribute information by Chao Game Representation (CGR) algorithm and disease attribute information by disease semantic similarity. Secondly, we extract feature by structural deep network embedding from the heterogeneous molecular associations network. Then, a comprehensive feature descriptor is constructed by combining attribute information and behavior information. Finally, Convolutional Neural Network (CNN) is adopted to train and classify these feature descriptors. In the five-fold cross validation experiment, SDNE-MDA achieved AUC of 0.9447 with the prediction accuracy of 87.38% on the HMDD v3.0 dataset. To further verify the performance of SDNE-MDA, we contrasted it with different feature extraction models and classifier models. Moreover, the case studies with three important human diseases, including Breast Neoplasms, Kidney Neoplasms, Lymphoma were implemented by the proposed model. As a result, 47, 46 and 46 out of top-50 predicted disease-related miRNAs have been confirmed by independent databases. These results anticipate that SDNE-MDA would be a reliable computational tool for predicting potential miRNA-disease associations.

## Introduction

MicroRNAs (miRNAs) are one type of small non-coding RNA with length of 20–25 nucleotides^1^. They normally influence their target messenger RNAs (mRNAs) by base pairing binding to the 3′ untranslated region (UTR) sites of mRNAs^2^. These small molecules could function as negative regulator of target gene expression in post-transcriptional^3^. With the development of molecular biology, increasing miRNAs have been detected^4^. To date, the famous miRbase database have collected 48,860 mature miRNAs from 271 organisms containing more than 1000 human miRNAs^5^. In addition, researchers have found that miRNAs are related with multiple significant cell biological activities, involving diffusion, aging, development, death and so on^6–9^.

In recent years, an increasing number of experiments have demonstrated that there are close relationships between miRNA with disease^10–13^. In particular, miRNAs have been new biomarkers for human cancer, which is important to cancer preventions and treatments^14^. Therefore, identifying the miRNA-disease associations has gradually become a hot topic in biology^15^. Early traditional biological experiments identified the disease-related miRNAs by detecting the expression level of miRNAs in biological disease process^16^. For example, Yohei et al*.* found that miR-200c could build a molecular link between breast cancer cells and normal cells^17^. Liu et al*.* point out that many miRNAs are disordered in cancer and this situation occurs because miRNAs participate in tumorigenesis and function as oncogenes^18^. Thum et al*.* reported that miR-21 adjust expression of the ERK-MAP kinase to effect on structure and function of heart^19^. Traditional experiments achieve high accuracy, while it has the limitations of long experimental time, high cost, and low success rate^20^. To resolve these issues, for effectively and accurately predict potential miRNA-disease associations, increasing researchers adopted computational model and select the most possible related miRNAs for further traditional biological experiments^21^.

With the development of biotechnology, some databases were constructed by collecting these biological data. These datasets provide the possibility to classify associations of miRNA-disease through computational methods^20,22–25^. Over the years, these methods mostly are according to the assumption that these functionally similar miRNAs tend to be related with semantically similar diseases^2,26–28^. These models could be split into under similarity network models and machine learning models^29^. For example, Jiang et al.^22^ presented a computational model to speculate the relationship between miRNA and disease based on a hypergeometric distribution model. This is an early calculation model by fusing multiple sources of information. However, this method built the miRNA-related network by functional similarity, which is limited by the relationship between miRNAs. Based on random walk method, Xuan et al.^30^ presented MIDP and MIDPE, an extension method of MIDP. MIDP constructed the network by combining the information of each node including similarity, prior information and various ranges of topological structure. This model could effectively reduce noise from data by restarting the walk. Furthermore, You et al.^31^ proposed PBMDA constructed a heterogeneous graph including three sub-graphs. PBMDA is a depth-first algorithm based on path, which could fully use the topology information of heterogeneous network. In particularly, the priority of new associations between diseases and miRNAs could be identified by evaluating the score of the path. Chen et al.^32^ proposed a computational method adopted the extreme gradient boosting named EGBMMDA. This is the first learning method based on decision tree for classifying miRNA-disease relationships. EGBMMDA built a comprehensive feature vector by various methods such as statistical, graph theory and matrix factorization. These studies have continually improved the performance of computational method and played an important guiding role in traditional biological experiments^33^. Therefore, accurately and effectively predict associations between miRNA-disease through computational method become urgently demanded^34^.

**Figure 1:**
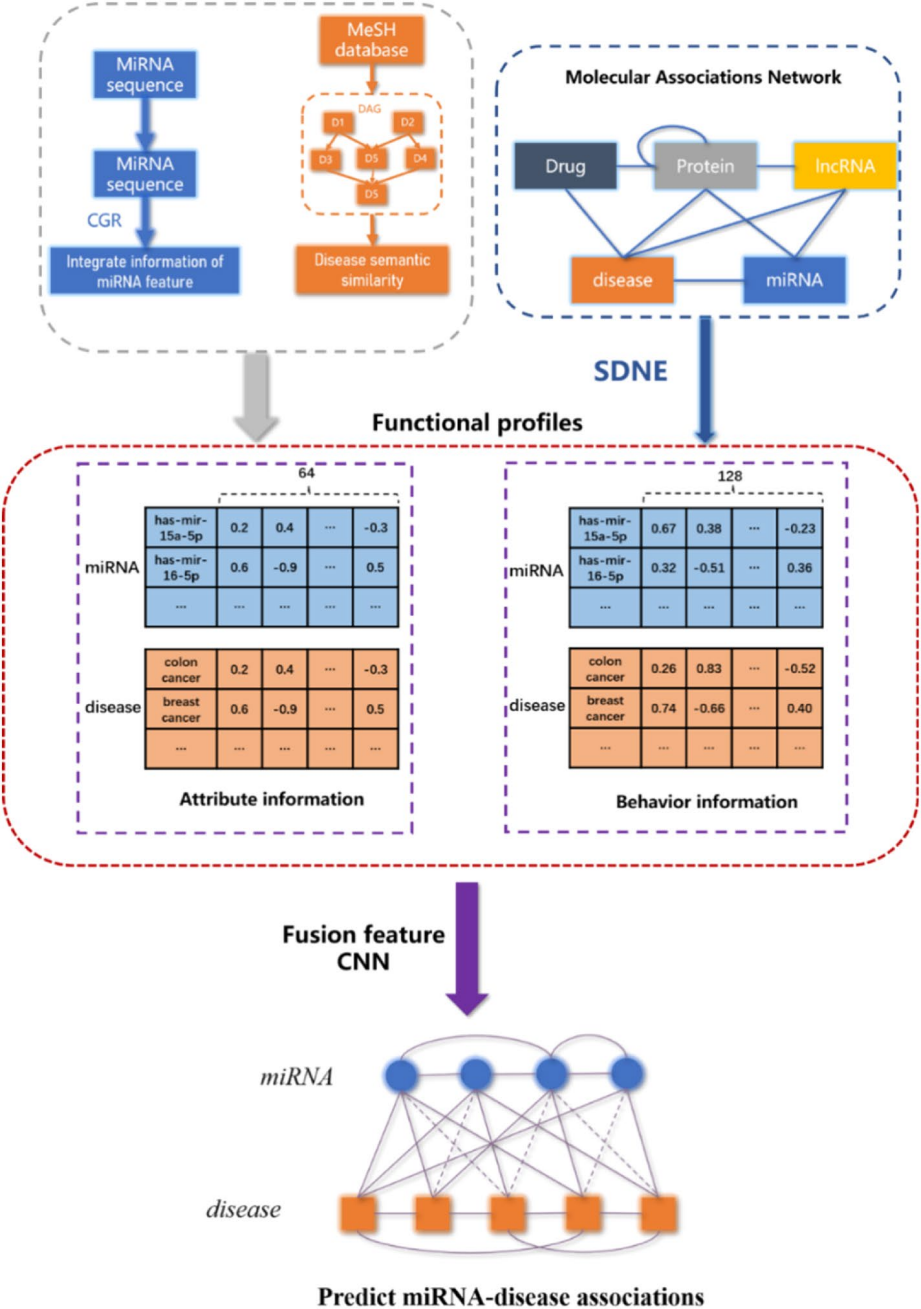
Flowchart of SDNE-MDA to predict potential miRNA-disease associations.

In this study, based on the assumption of molecules are related to each other in human physiological processes, we developed a structural deep network embedding-based model (SDNE-MDA) for predicting miRNA-disease association using molecular association network. The flow chart of SDNE-MDA is shown as Fig. 1. Specifically, we first constructed the molecular association network (MAN)^35^ by combining multiple different molecules with edges of them. This study extracted behavior information from the heterogeneous network by the structural deep network embedding (SDNE)^36^, which could maintain the overall structure of large network to the greatest extent. Secondly, SDNE-MDA obtained the miRNA attribute information by the chaos game representation (CGR) algorithm and disease attribute information by disease semantic similarity. After then, we formed the feature descriptor by fusing the behavior information and attribute information of miRNAs and diseases. Finally, these feature descriptors are trained and classified by the CNN to predict miRNA-disease associations. Five-fold cross validation experiment was carried out for SDNE-MDA to verify the performance of prediction and achieved the AUC of 0.9447 with the prediction accuracy of 87.38%. To further evaluate SDNE-MDA, we contrasted the proposed model with two feature extraction models and classifier models. Besides, we carry out SDNE-MDA with three significant human diseases involving breast cancer, kidney cancer and lymphoma. And as a result, 47, 46 and 46 out of top-50 candidate related miRNAs are confirmed by known databases and recent literature, respectively. These experiment result demonstrated that SDNE-MDA is a precisely and effectively computational method for predicting potential associations between miRNA with disease.

## Materials and methods

### Benchmark database

Human miRNA-disease associations benchmark database HMDD v3.0^37^ was adopted as data support in this paper, which collected 32,281 confirmed miRNA-disease associations, involving 1102 miRNAs and 850 diseases. Here, after data processing, we chose 16,427 known miRNA-disease associations as positive samples including 1023 miRNAs and 850 diseases. What’s more, we defined the adjacency matrix $AM$ to represent the miRNA-disease associations. When the miRNA $mi(a)$ have a verified association with the disease $di(b)$, we set $AM(mi(a),di(b))=1$, otherwise $AM(mi(a),di(b))=0$. In this paper, we introduce two other independent databases (dbDEMC^38^ and miR2Ddisease^39^) to verified the result of case study.

### Molecular associations network

**Figure 2:**
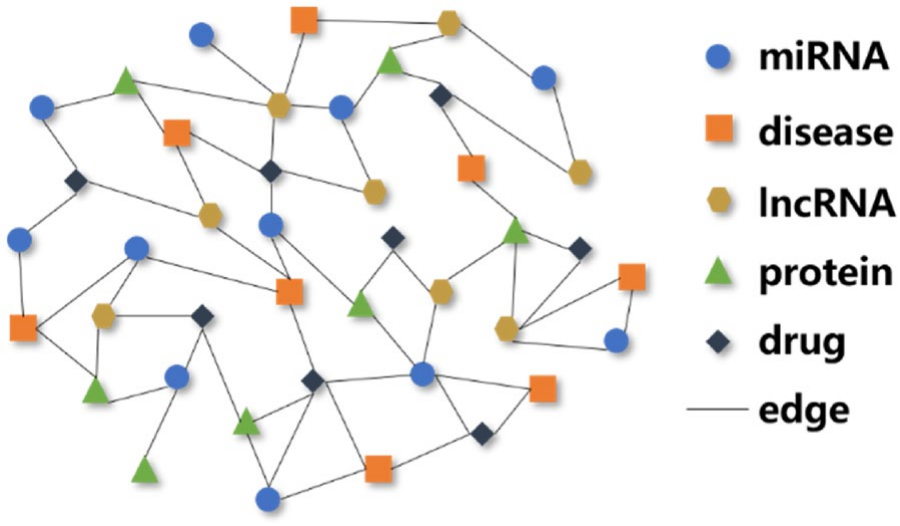
Structure diagram of molecular association network.

In this study, we combined multiple biological molecular information according the Molecular association network (MAN). The MAN is a heterogeneous information network proposed by Guo et al.^40^. Currently, this complex network consists of five types of molecular (miRNA, lncRNA, protein, disease, drug) and associations between them. The heterogeneous information network MAN provided a new comprehensive view to explore the complex physiological process and human disease. The structure diagram of molecular association network is as shown in Fig. 2. In this study, we download the information of molecular and associations between them from multiple databases. The number of different molecules is shown in Table 1, and the associations between them are shown in the following Table 2.

**Table 1:** The number of different types of nodes in MAN.

Molecular	Number
MiRNA	1023
Disease	2026
Drug	1025
LncRNA	769
Protein	1647
Total	6528

**Table 2:** The number and database of different types of associations in MAN.

Association	Database	Number
miRNA-disease	HMDD^41^	16,427
miRNA-protein	miRTarBase^42^	4944
Drug-protein	DrugBank^43^	11,107
lncRNA-disease	LncRNADisease^44^, LncRNASNP2^45^	1264
Protein–protein	STRING^46^	19,237
miRNA-lncRNA	lncRNASNP2^45^	8374
lncRNA-protein	LncRNA2Target^47^	690
Drug-disease	CTD^48^	18,416
Protein-disease	DisGeNET^49^	25,087
Total		105,546

### Chaos game representation (CGR) algorithm

MiRNA sequences contain a lot of complex information. However, most of the existing sequence feature information extraction algorithms only quantify one of position information and nonlinear information. In order to measure the similarity of these information contained in the miRNA sequences comprehensively. In this study, we chose chaos game representation (CGR)^50^ to quantize position and nonlinear information to calculate miRNA sequence similarity by pearson coefficient. Firstly, the positions of four nucleotides of miRNA are mapped to Euclidean space by the following formula:

1 $${T}_{i}={T}_{i-1}+c*\left({T}_{i-1}-{G}_{i}\right)$$

2 $$G_{i} = \left\{ {\begin{array}{*{20}l} {\left( {0,0} \right),} \hfill & {if\;type\;of\;nucleotide\;is\;A} \hfill \\ {\left( {0,1} \right),} \hfill & {if\;type\;of\;nucleotide\;is\;C} \hfill \\ {\left( {1,0} \right),} \hfill & {if\;type\;of\;nucleotide\;is\;U} \hfill \\ {\left( {1,1} \right),} \hfill & {if\;type\;of\;nucleotide\;is\;G} \hfill \\ \end{array} } \right.$$

where ${T}_{i}$ is the position of $i$th nucleotide, and it is related to the position of the previous nucleotide ${T}_{i-1}$ and the nucleotide coefficient ${G}_{i}$. In this paper, the contribution parameter $c$ is equal to 0.5 and ${T}_{0}$ is $(0.5, 0.5)$.

**Figure 3:**
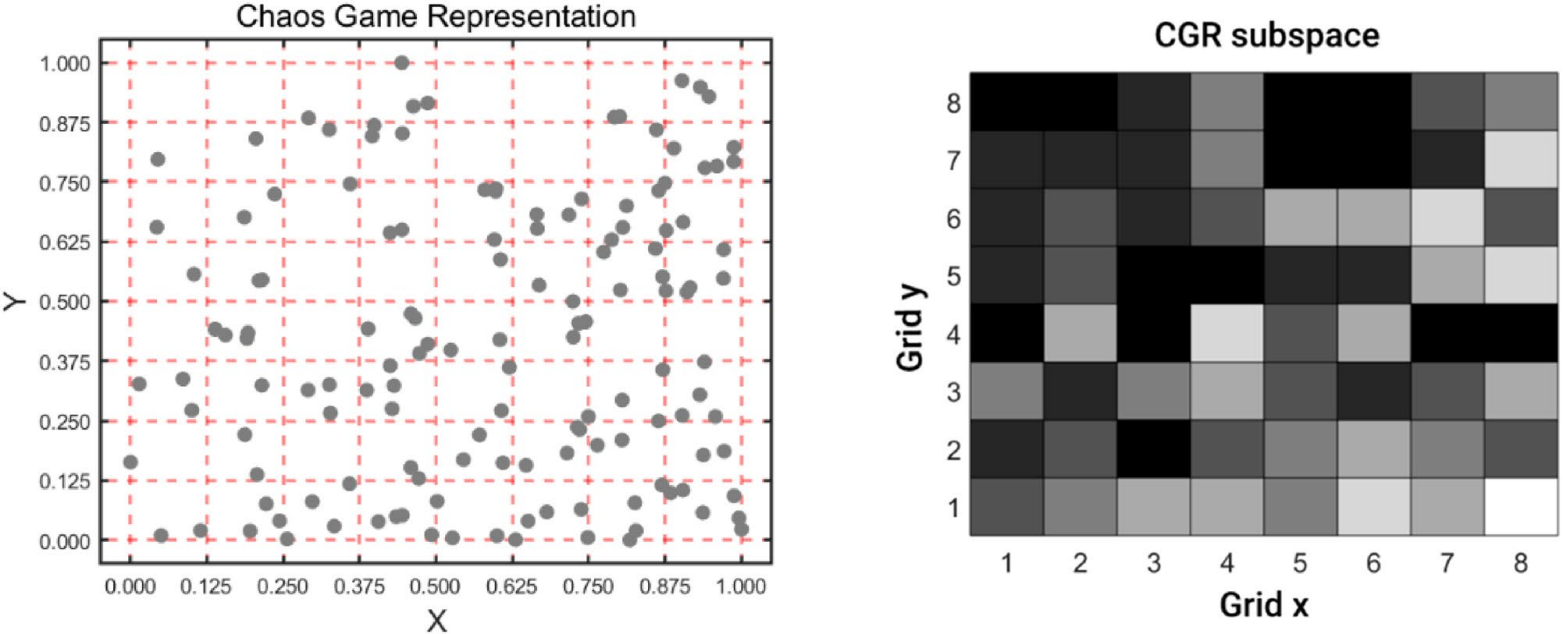
The CGR of has-mir-3976 plotted in $8\times 8$ subspaces and the matrix of its nucleotides with probabilities for chaos game representation.

Secondly, we divided the CGR space into 64 subspaces as shown in Fig. 3. The attribute information of each subspace ${SS}_{i}$ would be represented by integrating the position information ${X}_{i}, {Y}_{i}$ and nonlinear information ${Z}_{i}$ by the following formula:

3 $$X_{i} = \sum x ,\quad if\;point\;in\;subspace\;SS_{i}$$

4 $${Y}_{i}=\sum y, \quad if\;point\;in\;subspace\;{SS}_{i}$$

5 $${Z}_{i}=\frac{{num}_{i}-\frac{{\sum }_{t=1}^{64}{num}_{t}}{64}}{\sqrt{\frac{1}{64}{\sum }_{r=1}^{64}{({num}_{r}-\frac{{\sum }_{t=1}^{64}{num}_{t}}{64})}^{2}}}$$

6 $${SS}_{i}=\left({X}_{i},{Y}_{i},{Z}_{i}\right), i={1,2},\dots ,64$$

where ${num}_{i}$ is the number of points in subspace ${SS}_{i}$.

Finally, each miRNA sequence information could be represented by the descriptor $m(i)$. And we calculate sequence similarity ${M}_{sim}(m\left(i\right),m(j))$ by Pearson correlation coefficient.

7 $$m\left(i\right)=({SS}_{i},{SS}_{2},\dots ,{SS}_{64})$$

8 $${M}_{sim}\left(m\left(i\right),m\left(j\right)\right)=\frac{Cov(m\left(i\right),m(j))}{m\left(i\right)\times m(j)}$$

### Disease semantic similarity

In this study, the Directed Acyclic Graph (DAG)^51^ of diseases could be obtained from the Medical Subject Headings (Mesh)^52^. In the system, a disease $d(a)$ could be defined by $DAG(d(a)) = (L(d(a)), E(d(a)))$, where $L(d(a))$ is a node set including $d(a)$ and ancestor nodes of $d(a)$, and $E(d(a))$ indicates directed edge set of all relationships from ancestor node to child node. The semantic value of $d(a)$ was contributed by term $T$ as the formula:

9 $$\left\{ {\begin{array}{*{20}l} {D_{d\left( a \right)} \left( T \right) = 1} \hfill & {if\;T = d\left( a \right)} \hfill \\ {D_{d\left( a \right)} \left( T \right) = max\left\{ {\vartheta {*}D_{d\left( a \right)} \left( {T^{\prime}} \right)|T^{\prime} \in children\;of\;T} \right\}} \hfill & {if\;T \ne d\left( a \right)} \hfill \\ \end{array} } \right.$$

where $\vartheta$ is a parameter of semantic contribution, and $\vartheta$ is equal to 0.5 as previous study. Therefore, $DV\left(D\right)$ of $D$ could be calculated as follows:

10 $$DV\left(D\right)={\sum }_{T\in {A}_{D}}{D}_{D}(T)$$

According the assumption that two diseases should have higher similarity if they hold more same parts in DAG, the similarity of the diseases $d(a)$ with $d(b)$ could be obtained as follows:

11 $$S\left(d(a),d(b)\right)=\frac{\sum_{T\in {A}_{d(a)}\cap {A}_{d(b)}}({D}_{d(a)}\left(T\right)+{D}_{d(b)}(T))}{DV(d(a))+DV(d(b))}$$

### Structural deep network embedding

Since existing network embedding algorithms could not keep the high-order proximity of large-scale networks, this paper adopted the structural deep network embedding (SDNE) to extract the behavior information of miRNAs and diseases. Many existing network embedding models are shallow model (e.g. Laplacian Eigenmaps^53^, Graph Factorization^54^), which are unable to validly extract the highly non-linear structural information of network. SDNE is a semi-supervised model for network embedding. For the part of supervised, first-order similarity based on Laplacian matrix would be adopted to preserve local network information. And the part of unsupervised, SDNE used deep autoencoder modeling second-order similarity to save the global network information. Therefore, the loss function of SDNE is divided into two parts, i.e. Laplacian matrix model and Deep autoencoder model.

#### First-order similarity

To make adjacent nodes of graph closer in the latent space, the loss function of first-order similarity could be obtained as following formula:

12 $${L}_{1st}={\sum }_{i,j=1}^{n}{s}_{i,j}{\Vert {y}_{i}^{(k)}-{y}_{j}^{(k)}\Vert }_{2}^{2}={\sum }_{i,j=1}^{n}{s}_{i,j}{\Vert {y}_{i}-{y}_{j}\Vert }_{2}^{2}$$

where ${s}_{i,j}$ is the adjacency matrix for heterogeneous information network and ${y}_{i}^{(k)}$ indicates the node $i$ of $k$-th layer.

#### Second-order similarity

For the capturing of global structure information, SDNE construct the deep autoencoder model. Any given ${x}_{i}$ could be convert into the latent representation of $k$th layer as:

13 $${y}_{i}^{\left(1\right)}=\sigma \left({W}^{\left(1\right)}{x}_{i}+{b}^{\left(1\right)}\right)$$

14 $${y}_{i}^{\left(k\right)}=\sigma \left({W}^{\left(k\right)}{y}_{i}^{\left(k-1\right)}+{b}^{\left(k\right)}\right), k=2,\dots , K$$

here ${W}^{\left(k\right)}$ is the $k$th layer weight matrix and ${b}^{\left(k\right)}$ as a parameter. According the optimization goal of the autoencoder is to reduce the reconstruction error in input and output, therefore, we could define the loss function as follows:

15 $$L={\sum }_{i=1}^{n}{\Vert \widehat{{x}_{i}}-{x}_{i}\Vert }_{2}^{2}$$

The adjacency matrices are often very sparse, which means zero elements are far more than non-zero elements. Therefore, the loss function would be optimized as:

16 $${L}_{2{\text{nd}}}={\sum }_{i=1}^{n}{\Vert (\widehat{{x}_{i}}-{x}_{i})\odot {b}_{i}\Vert }_{2}^{2}={\Vert (\widehat{X}-X)\odot B\Vert }_{\text{F}}^{2}$$

where $\odot$ is the Hadamard product (multiplying the corresponding elements).

Integrating the first-order similarity and second-order similarity, the finally loss function of SDNE is shown as follows:

17 $${L}_{mix}={L}_{2nd}+{\upalpha }{L}_{1st}+\upsilon {L}_{reg}={\Vert (\widehat{X}-X)\odot B\Vert }_{\text{F}}^{2}+\alpha {\sum }_{i,j=1}^{n}{s}_{i,j}{\Vert {y}_{i}-{y}_{j}\Vert }_{2}^{2}+\upsilon {L}_{reg}$$

where ${L}_{reg}$ is a regularization term, and $\alpha$ is a parameter to control the loss of the first-order similarity. The regularization term is shown as:

18 $$L_{reg} = \frac{1}{2}\sum\limits_{k = 1}^{K} {\left( {W_{F}^{\left( k \right)2} + \hat{W}_{F}^{\left( k \right)2} } \right)}$$

### Integration of feature information

In this study, we firstly obtained miRNA sequence similarity and disease semantic similarity and convert them into attribute feature information ${M}_{sim}(i)$, ${D}_{sim}(j)$ of same dimension by stacked autoencoder. The dimension of ${M}_{sim}(i)$ and ${D}_{sim}(j)$ is 64. After then, the behavior feature information of miRNAs ${M}_{b}(i)$ and diseases ${D}_{b}(j)$ were extracted by the structural deep network embedding based on the molecular association network. The dimension of ${M}_{b}(i)$ and ${D}_{b}(j)$ is 128. Finally, a complete sample feature descriptor is constructed by fusing above information based on the HMDD v3.0 database. The feature descriptor was a 384-dimensional vector as follows:

19 $$FD\left(i,j\right)=\left[{M}_{b}\left(i\right),{M}_{sim}\left(i\right),{D}_{b}\left(j\right),{D}_{sim}\left(j\right)\right]$$

### Convolutional neural network algorithm

**Figure 4:**
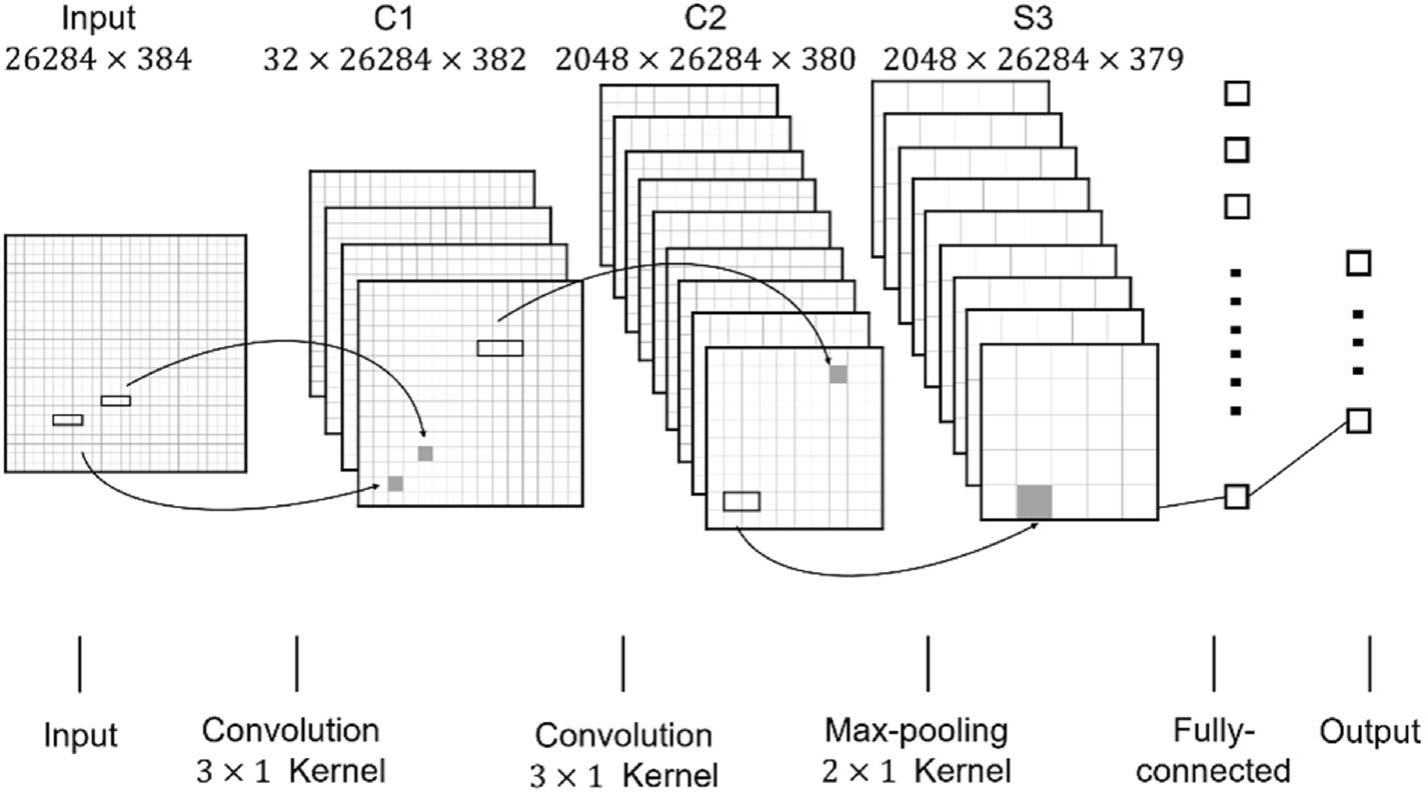
Structure of the CNN algorithm.

Convolutional neural network (CNN) is a deep-structured feedforward neural network with convolution calculations. CNN could shift-invariant classify the input information based on layer structure by representation learning capability. With the development of research, CNN has been successfully utilized in bioinformatics^55^. Therefore, in this paper, we adopted the CNN to train and predict potential miRNA-disease association. Specifically, CNN has a multi-layer structure including input, convolutional layer, pooling layer, fully-connected layer and output as shown in Fig. 4. The input layer is a matrix of all feature descriptor $FD\left(i,j\right)$ with size $26284\times 384$. Two convolutional layers $C1$ and $C2$ are obtained by 32 filters with $3\times 1$ convolution kernel and 64 filters with $3\times 1$ convolution kernel. In this study, we adopted max-pooling $2\times 1$ kernel to subsample the $C2$. After repeatedly convolution and pooling, CNN classifies the features from fully-connected layer and output the probability distribution.

## Results and discussion

### Performance evaluation

**Figure 5:**
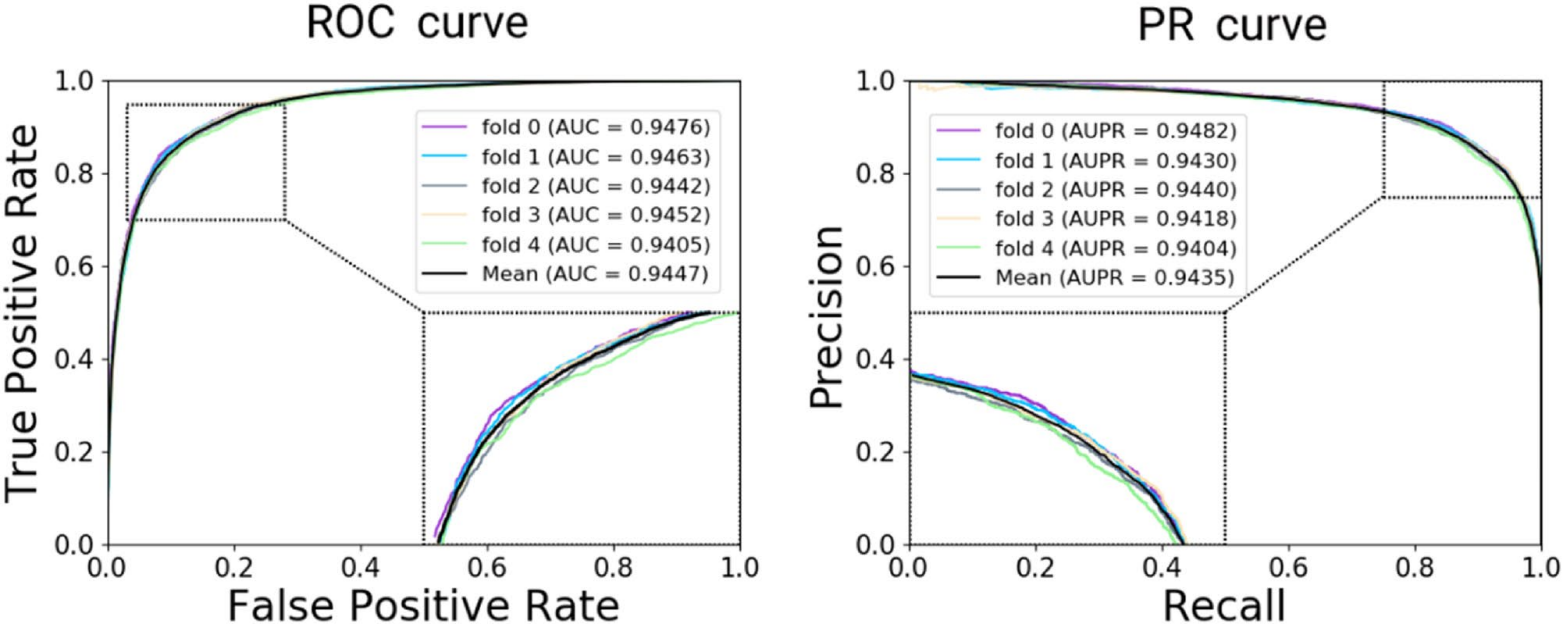
The ROC and PR curves performed in terms of five-fold cross validation by SDNE-MDA on HMDD v3.0.

In this experiment, we implemented the five-fold cross validation to evaluate the performance of proposed model under HMDD v3.0^37^. These known miRNA-disease pairs would be randomly split into five subsets with no intersection. Each cross validation, one of five subsets would be set as test set and remaining data sets as train set. To avoid the revelation of test data, we constructed the heterogeneous information network by only training data and extract the behavior information. In this study, a class of evaluation criteria were used to assess SDNE-MDA, including accuracy (Acc.), sensitivity (Sen.), specificity (Spec.), precision (Prec.), Matthews Correlation Coefficient (MCC) and area under curve (AUC). As a result, the average Acc, Sen, Spec, Prec, MCC and AUC achieved 87.38%, 87.28%, 87.47%, 87.45%, 74.76% and 0.9447 with standard deviations of 0.44%, 0.93%, 1.01%, 0.82%, 0.88% and 0.0027, respectively as shown in Table 3. In addition, the receiver operating characteristics (ROC) curve and area under precision-recall (PR) curve by SDNE-MDA based on HMDD are shown in Fig. 5.

**Table 3:** Five-fold cross validation results performed by SDNE-MDA on HMDD v3.0.

Evaluation criteria	Result
Acc. (%)	87.38 ± 0.44
Sen. (%)	87.28 ± 0.93
Spec. (%)	87.47 ± 1.01
Prec. (%)	87.45 ± 0.82
MCC (%)	74.76 ± 0.88
AUC	0.9447 ± 0.0027

### Comparison with different feature extraction methods

**Figure 6:**
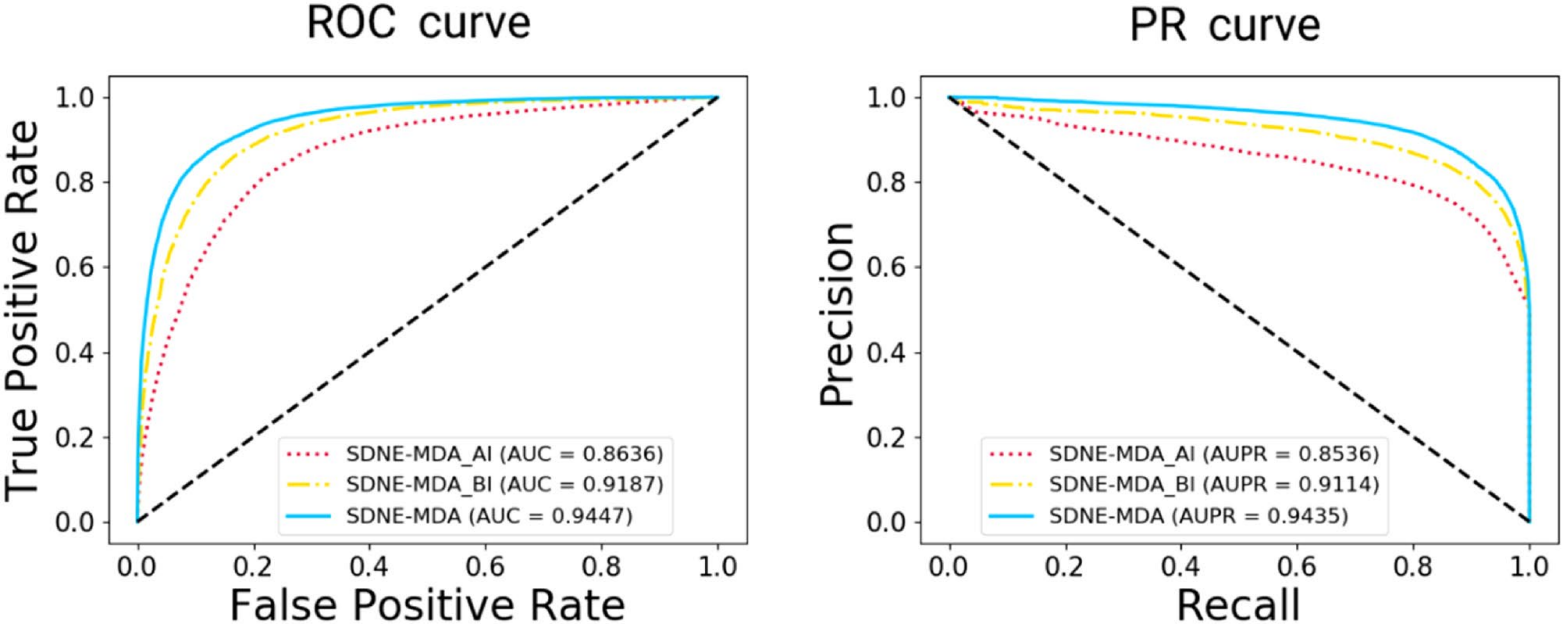
ROC and PR curves performed by SDNE-MDA_AI, SDNE-MDA_BI and SNDE-MDA model in terms of five-fold cross validation based on HMDD database.

In this study, these nodes in the network could be represented by the attribute and behavior information. Both types of information may influence the result of prediction, so we compared the different feature extraction methods including SDNE-MDA_AI composed of attribute information, SDNE-MDA_BI composed of behavior information and SDNE-MDA composed of both them. In addition, attribute information of other nodes has scarcely effect on prediction of potential miRNA-disease relationships. For reducing the redundancy of model, we only considered the attribute information of miRNAs and diseases. The detail result of comparison between proposed model with different feature extraction models are shown in Table 4. The accuracy of SDNE-MDA is 7.78% and 3.43% higher than that of SDNE-MDA_AI and SDNE-MDA_BI, respectively. In addition, the AUC of proposed model is 0.0811 and 0.0260 higher than SDNE-MDA_AI and SDNE-MDA_BI. The ROC curves and PR curves of three experiments are shown in Fig. 6. These results indicated that integrating the two kind of information to represent the node achieved more distinguished performance.

**Table 4:** The comparison results between SDNE-MDA_AI model, SDNE-MDA_BI model and SDNE-MDA model based on HMDD database.

Feature	Acc. (%)	Sen. (%)	Spec. (%)	Prec. (%)	MCC (%)	AUC
SDNE-MDA_AI	79.60 ± 0.35	81.29 ± 1.87	77.92 ± 1.43	78.65 ± 0.73	59.26 ± 0.73	0.8636 ± 0.0037
SDNE-MDA_BI	83.95 ± 0.72	83.08 ± 6.30	84.83 ± 5.94	84.95 ± 4.07	68.32 ± 1.27	0.9187 ± 0.0048
SDNE-MDA	87.38 ± 0.44	87.28 ± 0.93	87.47 ± 1.01	87.45 ± 0.82	74.76 ± 0.88	0.9447 ± 0.0027

### Comparison with different classifier models

**Figure 7:**
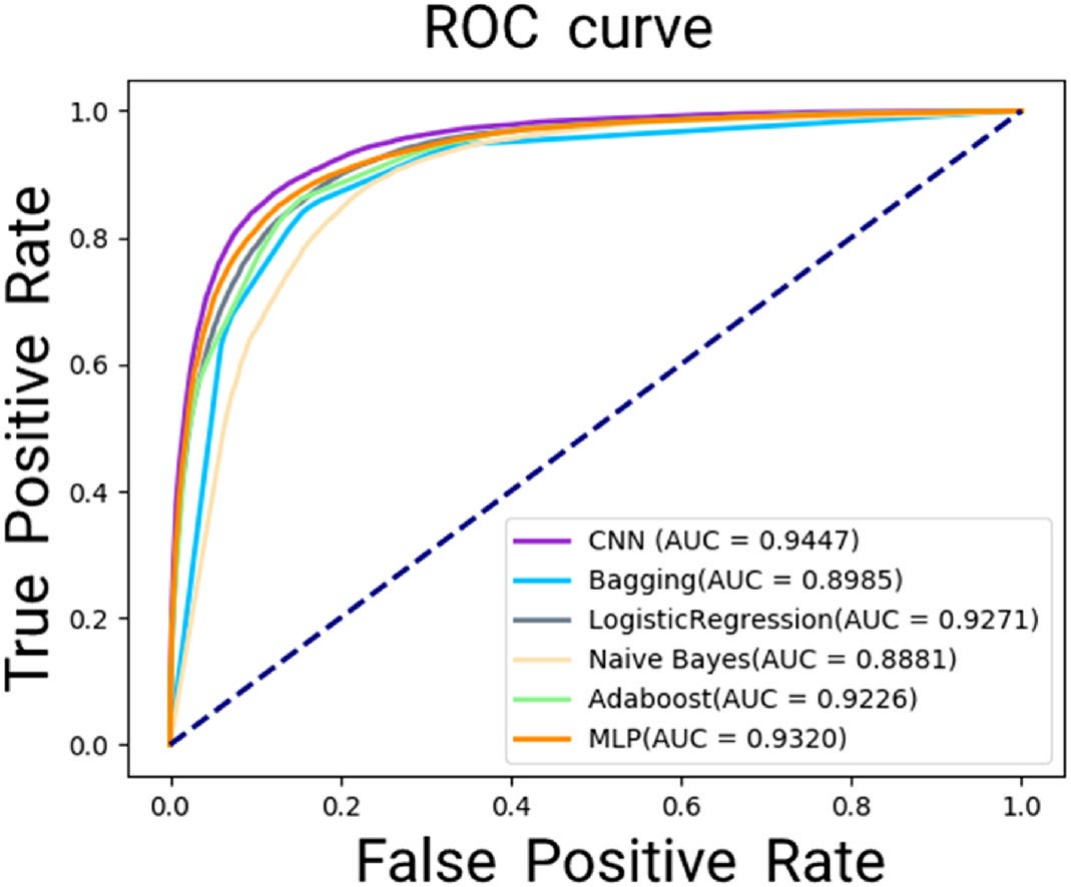
Performance comparison between SDNE-MDA with other four different classifier models based on HMDD v3.0 database.

In this study, the CNN was adopted to train and identify potential relationships between miRNA and disease. To further evaluate SDNE-MDA, we compare proposed model with Bagging, Logistic Regression, Naive Bayes and Adaboost classifier model. In this experiment, we implemented the five-fold cross validation in these different classifier models based on the HMDD v3.0. Finally, the proposed model yielded average AUC of 0.9447 based on five-fold cross validation and outperformed Bagging (0.8998), LogisticRegression (0.9270), Naive Bayes (0.8881), Adaboost (0.9226) and MLP (0.9320). The AUC of CNN is 0.0259 higher than the mean AUC of all five model, and the accuracy is 1.60% higher than that of the second highest methods. The detail results of the comparison between SDNE-MDA and other four classifier models are shown in Table 5, and we drew the ROC curves as shown in Fig. 7. Therefore, CNN algorithm is the optimal selection for the proposed model to predicting potential miRNA-disease associations.

**Table 5:** The comparison results between SDNE-MDA with other four different classifier models in terms of five-fold cross validation based on HMDD v3.0 database.

Model	Acc. (%)	Sen. (%)	Spec. (%)	MCC (%)	AUC
SDNE-MDA	87.38 ± 0.44	87.28 ± 0.93	87.47 ± 1.01	74.76 ± 0.88	0.9447 ± 0.0027
Bagging	84.52 ± 0.62	$84.77\hspace{0.17em}\pm \hspace{0.17em}$0.80	84.27 ± 1.34	69.05 ± 1.23	0.8985 ± 0.0042
LogisticRegression	85.13 ± 0.86	84.42 ± 0.92	85.84 ± 1.19	70.27 ± 1.71	0.9272 ± 0.0080
NaiveBayes	75.90 ± 1.27	60.04 ± 3.94	91.76 ± 1.64	54.68 ± 1.77	0.8881 ± 0.0059
Adaboost	85.69 ± 0.51	84.74 ± 1.72	86.63 ± 2.07	71.43 ± 1.06	0.9226 ± 0.0036
MLP	85.78 ± 1.06	84.75 ± 0.87	86.82 ± 2.78	71.72 ± 1.83	0.9320 ± 0.0051

### Comparison with related work

An increasing number of researchers have focused on the prediction of miRNA-disease associations, and a mass of model have been proposed. To further evaluate the predictive performance of our method, the SDNE-MDA was compared with six state-of-the-art classical methods under five-fold cross validation, including RWRMDA^56^, MTDN^57^, EGBMMDA^32^, LMTRDA^58^, DBMDA^59^ and PBMDA^31^. Since these algorithms have not calculated multiple evaluation criteria, we only compare the AUC on the terms of five-fold cross validation based HMDD database. The detail results of the comparison between SDNE-MDA and other six related works are shown in Table 6. The proposed method is 0.0399 higher than the average AUC of all algorithms, and 0.0275 higher than that of the second highest methods. This is mainly due to SDNE-MDA integrated two types of information of miRNAs and diseases, and extract the feature more comprehensively. Therefore, the proposed model is an effective and reliable computational tool for predicting potential miRNA-disease associations.

**Table 6:** The comparison results between SDNE-MDA with other related works.

Method	AUC
RWRMDA	0.8617
MTDN	0.8872
EGBMMDA	0.9048
LMTRDA	0.9054
DBMDA	0.9129
PBMDA	0.9172
SDNE-MDA	0.9447

### Case studies

For further evaluating the prediction ability of SDNE-MDA, we implemented case studies based on three significant human diseases (Breast Neoplasms, Kidney Neoplasms, Lymphoma). In this study, these known miRNA-disease associations based on HMDD v3.0 database would be the training set. To avoid the overlap in the train data and prediction list, the test set is the unknown relationship pairs between three diseases and all possible miRNAs. As a result, 47, 46 and 46 of top-50 candidate related miRNAs were confirmed by independent databases. Therefore, SDNE-MDA is a feasible and reliable model for predicting potential relationships between miRNA and disease.

Breast Neoplasms is the most universal neoplasms in female and the risk of breast cancer is up to 13% in the United States. Although men may also develop breast cancer, 99% of patients are women. There are approximately 276,480 novel cases in women and 42,170 were die from breast cancer in 2020^60^. In previous few years, studies had indicated the expression level of miRNA have strong impact to growth and division of breast tumor cell^61^. Therefore, we implemented a case study of Breast Neoplasms-miRNA associations by SDNE-MDA. In the prediction list shown as Table 7, 47 of top 50 predicted Breast Neoplasms related miRNAs were verified based on independent databases.

**Table 7:** Prediction of top 50 miRNAs related to Breast Neoplasms based on known miRNA-disease associations in HMDD V3.0 database.

Rank	miRNA	Evidence	Rank	miRNA	Evidence
1	hsa-miR-124-3p	dbdemc	26	hsa-miR-200b-3p	dbdemc
2	hsa-miR-483-5p	dbdemc	27	hsa-miR-181d-5p	dbdemc
3	hsa-miR-200c-3p	dbdemc	28	hsa-miR-23b-3p	dbdemc
4	hsa-miR-101-3p	dbdemc	29	hsa-miR-532-5p	dbdemc
5	hsa-miR-27a-3p	dbdemc	30	hsa-miR-193b-3p	dbdemc
6	hsa-miR-28-5p	dbdemc	31	hsa-miR-126-3p	dbdemc
7	hsa-miR-455-5p	dbdemc	32	hsa-miR-92b-3p	dbdemc
8	hsa-miR-186-5p	dbdemc	33	hsa-miR-539-5p	dbdemc
9	hsa-miR-99b-5p	dbdemc	34	hsa-mir-138-2-3p	Unconfirmed
10	hsa-miR-141-3p	dbdemc	35	hsa-miR-506-3p	dbdemc
11	hsa-miR-330-5p	dbdemc	36	hsa-miR-223-3p	dbdemc
12	hsa-miR-19b-2-5p	dbdemc	37	hsa-miR-19a-3p	dbdemc
13	hsa-miR-154-5p	dbdemc	38	hsa-miR-29c-3p	dbdemc
14	hsa-miR-744-5p	dbdemc	39	hsa-miR-188-5p	dbdemc
15	hsa-miR-1271-5p	dbdemc	40	hsa-miR-25-3p	dbdemc
16	hsa-miR-377-3p	dbdemc	41	hsa-miR-300	dbdemc
17	hsa-miR-200a-3p	dbdemc	42	hsa-miR-376b-3p	dbdemc
18	hsa-miR-211-5p	dbdemc	43	hsa-mir-208b-5p	Unconfirmed
19	hsa-miR-216a-5p	dbdemc	44	hsa-miR-376a-3p	dbdemc
20	hsa-miR-449b-5p	dbdemc	45	hsa-miR-543	dbdemc
21	hsa-miR-346	dbdemc	46	hsa-miR-130a-3p	dbdemc
22	hsa-miR-328-3p	dbdemc	47	hsa-miR-302a-3p	dbdemc
23	hsa-miR-494-3p	dbdemc	48	hsa-miR-29a-3p	dbdemc
24	hsa-mir-885-5p	Unconfirmed	49	hsa-miR-302e	dbdemc
25	hsa-miR-202-3p	dbdemc	50	hsa-miR-363-3p	dbdemc

Kidney Neoplasms is a novel cancer with higher adult incidence^60^. In the past few years, however, morbidity and mortality of kidney neoplasms have been increasing. There are about 73,750 novel cases in kidney neoplasms with about 45,520 in male and about 28,230 in female in United States and about 14,830 deaths for this cancer (9860 men and 4970 women) in 2020. Recently, increasing researchers have indicated miRNAs are related with kidney neoplasms^62^. Thus, we take Kidney Neoplasms as a case study for SDNE-MDA and prioritize the candidate miRNAs. In the prediction list shown as Table 8, 46 of top-50 potential kidney neoplasms-related miRNAs were confirmed by independent databases.

**Table 8:** Prediction of top 50 miRNAs related to Kidney Neoplasms based on known miRNA-disease associations in HMDD V3.0 database.

Rank	miRNA	Evidence	Rank	miRNA	Evidence
1	hsa-mir-146a-5p	dbdemc	26	hsa-mir-19a-5p	dbdemc
2	hsa-mir-223-5p	dbdemc	27	hsa-mir-133a-5p	Unconfirmed
3	hsa-mir-125b-5p	dbdemc	28	hsa-mir-29b-3p	dbdemc
4	hsa-mir-145-5p	dbdemc	29	hsa-mir-222-5p	dbdemc
5	hsa-mir-150-5p	dbdemc	30	hsa-mir-29c-5p	dbdemc
6	hsa-mir-181a-5p	dbdemc	31	hsa-mir-18a-5p	dbdemc
7	hsa-mir-182-5p	dbdemc	32	hsa-mir-1-3p	dbdemc
8	hsa-mir-26a-5p	dbdemc	33	hsa-mir-181b-5p	dbdemc
9	hsa-mir-9-5p	dbdemc	34	hsa-mir-206	dbdemc
10	hsa-mir-31-5p	dbdemc	35	hsa-mir-124-5p	Unconfirmed
11	hsa-mir-16-5p	dbdemc	36	hsa-mir-205-5p	Unconfirmed
12	hsa-mir-143-5p	dbdemc	37	hsa-mir-23a-5p	dbdemc
13	hsa-mir-221-5p	dbdemc	38	hsa-let-7c-5p	dbdemc
14	hsa-mir-20a-5p	dbdemc	39	hsa-mir-22-5p	dbdemc
15	hsa-mir-26b-5p	dbdemc	40	hsa-mir-34b-5p	dbdemc
16	hsa-let-7b-5p	dbdemc	41	hsa-mir-19b-3p	dbdemc
17	hsa-mir-92a-3p	dbdemc	42	hsa-mir-132-5p	dbdemc
18	hsa-mir-29a-5p	dbdemc	43	hsa-mir-106b-5p	dbdemc
19	hsa-mir-375-5p	Unconfirmed	44	hsa-mir-34c-5p	dbdemc
20	hsa-mir-142-5p	dbdemc	45	hsa-mir-100-5p	dbdemc
21	hsa-let-7a-5p	dbdemc	46	hsa-mir-124-3p	dbdemc
22	hsa-mir-122-5p	dbdemc	47	hsa-mir-125a-5p	dbdemc
23	hsa-mir-146b-5p	dbdemc	48	hsa-mir-148a-5p	dbdemc
24	hsa-mir-30a-5p	dbdemc	49	hsa-mir-200b-5p	dbdemc
25	hsa-mir-24-3p	dbdemc	50	hsa-mir-486-5p	dbdemc

Lymphoma is one of the most common malignant cancers (~ 4% of all new cancer) especially in teenagers in United States^60^. Lymphoma mainly contains two types of Hodgkin Lymphoma (HL) and non-Hodgkin Lymphoma (NHL). In 2020, it is estimated that about 85,720 new cases of Lymphoma (47,070 of men and 38,650 of women) and 20,910 deaths for HL and NHL (12,030 of men and 8,880 of women). Therefore, we implemented SDNE-MDA to prioritize possible miRNAs for Lymphoma based on HMDD v3.0. As shown in Table 9, 46 out of top 50 predicted Lymphoma candidate miRNAs were verified by independent databases.

**Table 9:** Prediction of top 50 miRNAs related to Lymphoma based on known miRNA-disease associations in HMDD V3.0 database.

Rank	miRNA	Evidence	Rank	miRNA	Evidence
1	hsa-mir-34a-5p	dbdemc	26	hsa-mir-138-5p	dbdemc
2	hsa-mir-223-5p	dbdemc	27	hsa-mir-106a-5p	dbdemc
3	hsa-mir-125b-5p	dbdemc	28	hsa-mir-34b-5p	dbdemc
4	hsa-mir-145-5p	dbdemc	29	hsa-mir-140-5p	dbdemc
5	hsa-mir-182-5p	dbdemc	30	hsa-mir-132-5p	dbdemc
6	hsa-mir-27a-5p	Unconfirmed	31	hsa-mir-106b-5p	dbdemc
7	hsa-mir-9-5p	dbdemc	32	hsa-mir-100-5p	dbdemc
8	hsa-mir-26b-5p	dbdemc	33	hsa-mir-34c-5p	dbdemc
9	hsa-let-7b-5p	dbdemc	34	hsa-mir-148a-5p	dbdemc
10	hsa-mir-29a-5p	dbdemc	35	hsa-mir-124-3p	dbdemc
11	hsa-let-7a-5p	dbdemc	36	hsa-mir-25-5p	dbdemc
12	hsa-mir-192-5p	dbdemc	37	hsa-let-7i-5p	dbdemc
13	hsa-mir-146b-5p	dbdemc	38	hsa-mir-335-5p	dbdemc
14	hsa-mir-30a-5p	dbdemc	39	hsa-mir-141-5p	Unconfirmed
15	hsa-mir-24-3p	dbdemc	40	hsa-mir-99a-5p	dbdemc
16	hsa-mir-214-5p	dbdemc	41	hsa-mir-107	dbdemc
17	hsa-mir-96-5p	dbdemc	42	hsa-mir-15b-5p	dbdemc
18	hsa-mir-183-5p	dbdemc	43	hsa-mir-144-5p	dbdemc
19	hsa-mir-206	dbdemc	44	hsa-let-7e-5p	dbdemc
20	hsa-mir-181b-5p	dbdemc	45	hsa-mir-30d-5p	dbdemc
21	hsa-mir-1-3p	dbdemc	46	hsa-mir-218-5p	dbdemc
22	hsa-let-7c-5p	dbdemc	47	hsa-mir-130a-5p	Unconfirmed
23	hsa-mir-205-5p	dbdemc	48	hsa-mir-429	Unconfirmed
24	hsa-mir-124-5p	dbdemc	49	hsa-mir-101-5p	dbdemc
25	hsa-mir-23a-5p	dbdemc	50	hsa-mir-195-5p	dbdemc

## Conclusion

In previous few years, accumulating number of researches demonstrated that miRNAs have closely link with diseases. Various of biological experiments and computational methods are committed to classify the association of them. In this paper, we proposed a structural deep network embedding-based model SDNE-MDA to predict miRNA-disease associations. This model constructed a complex network MAN by fusing miRNAs, diseases and three related molecular (lncRNA, drug and protein) with their relationships. Through the comprehensive heterogeneous information network, potential miRNA-disease associations could be predicted more accurate and efficient. And CNN is utilized to train and classify the potential miRNA-disease associations. Compared with other classifiers and feature extraction models, SDNE-MDA showed outstanding performance. In addition, case studies were implemented on three significant human disease for further validate performance of SDNE-MDA. As a result, 47, 46 and 46 of top-50 predicted miRNAs have been confirmed by independent databases. These results demonstrated that SDNE-MDA is a reliable computational tool for predicting miRNA-disease associations.
